# Solving the spectral gap problem with a four-mirror bow-tie cavity in waveguide free-electron laser oscillators

**DOI:** 10.1038/s41598-023-49803-w

**Published:** 2023-12-19

**Authors:** Mengqi Xia, Yuanfang Xu, Zhouyu Zhao, Heting Li

**Affiliations:** National Synchrotron Radiation Laboratory, University of Science and Technology of China, Hefei, 230029 China

**Keywords:** Free-electron lasers, Terahertz optics, Optical physics

## Abstract

The generation of intense coherent radiation pulses in the far-infrared and terahertz regimes is of considerable interest to the free-electron laser (FEL) radiation user community. At long wavelengths, the diffraction effect can be quite severe, therefore, an optical waveguide is required to confine the radiation field. However, it will also bring about some new phenomena, and the most noteworthy one is the spectral gap phenomenon: at some particular wavelengths, regardless of electron beam adjustments, the coupling efficiency and output power of waveguide FEL oscillators drop significantly. Such spectral gap has an adverse effect on experimental results since numerous experiments require continuous spectral scanning. In this paper, we propose to utilize a bow-tie cavity instead of conventional cavities to the waveguide FEL to solve the spectral gap problem. The simulation was carried out based on the parameters of FELiChEM, a newly built user facility in China. Numerical simulation code OPC combining with modified GENESIS is used to enable the modelling, for the first time, of a bow-tie cavity based FEL in the far-infrared wavelength regime. The simulation results indicate that this novel structure can effectively eliminate the spectral gaps and substantially enhance long-wavelength laser performance.

## Introduction

Free Electron Lasers (FELs) are powerful, tunable, and versatile radiation sources used in various scientific and industrial applications, spanning from microwaves to hard X-rays^1^. Several FEL facilities have been developed or built around the world with wavelength ranges covering the hard X-ray to THz spectrum^2,3^. FELs can operate as high-gain, single-pass amplifiers or low-gain oscillators. In the latter case, the FEL undulator is installed within a cavity to provide radiation feedback. The free electron laser oscillator (FELO) is one of the main operation modes in current FELs, especially suitable for the infrared and terahertz range. There are a number of FELO facilities that are built worldwide as test/user facilities, such as CLIO^4^, FELIX^5^, ELBE^6^, JAERI^7^, KU-FEL^8^, FHI-FEL^9^, FLARE^10^, FELiChEM^11^ and so on. These FEL devices operate in the THz band, characterized by relatively long wavelengths in the radiation field. However, as the wavelength increases, the diffraction effect becomes more severe. The waist of a confocal resonator for infrared radiation produced by a FELO typically exceeds the undulator gap, therefore an optical waveguide is required.

With the optical waveguide, several schemes have been developed over the past few decades to improve FELO properties. According to the extent of waveguide coverage within the resonant cavity, the waveguide FEL can be divided into full-cavity waveguide mode, partial-cavity waveguide mode and hybrid cavity waveguide mode. In the full-cavity waveguide FEL, the waveguide covers all the space in which the light field passes. The FLARE^10^ device, for example, uses a full-cavity waveguide mode, which has a strong waveguide effect in the resonant cavity and a complex optical field mode. Partial-cavity waveguide is a mode in which the waveguide is only present in the undulator and the waveguide length is equal to the undulator length. The light field propagates through the rest of the cavity in free-space mode. The CLIO^12^ and FELiChEM^11^ devices operate in this mode. The advantage of this technology lies in its relatively simple optical field modes within the cavity as well as its ease of implementation in structural engineering. The hybrid waveguide mode falls between the two aforementioned states. In general, its coverage extends from the upstream cavity mirror or downstream cavity mirror to the opposite end of the undulator, such as FELIX^5^ and ELBE^6^.

However, the waveguide also gives rise to some new effects on FEL performance. The most noticeable difference between the waveguide FEL and its free-space counterpart is the frequency dependence of the gain. In an FELO, an output coupling hole with a diameter of several millimeters is located in the center of a cavity mirror. The advantage of the hole-coupling scheme is that it does not create optical absorption, unlike a beam splitter. Nevertheless, the output coupling factor of the hole, i.e., the ratio between output power and intracavity power, is strongly affected by the transverse distribution of laser mode. Generally speaking, the waveguide effects depend on the waveguide parameters. When the waveguide length exceeds a certain threshold, ‘mode disorder’ occurs in the resonant cavity, which makes the laser difficult to work in order^13^. In the waveguide FELs, the most common and obvious phenomenon is the spectral gap phenomenon, which implies that the output power is greatly reduced in some wavelength bands. It will lead to laser power discontinuity in spectral scanning, which is usually unavoidable within a broad wavelength range. At present, numerous FELO devices commonly exhibit a substantial presence of spectral gaps in the long wavelength range, leading to significant limitations in spectral scanning experiments for users.

As the electromagnetic wave propagates within the inner space of the waveguide, typically a hollow metal tube in an FELO, its movement is constrained by the metal pipe wall serving as the boundary. Considering both waveguide and free space conditions, the longitudinal dependence of the electron beam and radiation field, including the resonant frequency and group velocity, is approximately the same^14^. Their main difference is the dependence of the transverse field evolution on the waveguide. The radiation field can be decomposed into a large number of eigenfunctions, where each eigenmode has a different propagation speed in the waveguide. According to Prazeres et al.’s research^12,15^, the occurrence of the spectral gap phenomenon primarily results from the unique combination of characteristic modes $\text {TE}_{\text{q}}$ and $\text {TM}_{\text{q}}$ induced by the waveguide. In the waveguide FEL, different modes *q* propagate through the waveguide at different speeds, leading to phase changes and distribution deformation of the corresponding radiation field in the resonant cavity, which subsequently affects laser output energy. A more general formula is developed by Prazeres et al.^12^ to show the wavelength positions of spectral gap in the waveguide FEL:

$$\lambda =\frac{4(2 n-1)}{q_{2}^{2}-q_{1}^{2}} \frac{b^{2}}{L} \quad \left( n = N_{+}\right)$$

where *L* is the length of the waveguide and $\left( 2n-1 \right) \pi$ represents the phase difference between the mode order $q_{2}$ and $q_{1}$. Generally, the horizontal size *a* of a rectangular waveguide is larger than the vertical size *b*, which has little effect on the spectral gap. The wavelength positions of spectral gap in the waveguide FEL is mainly influenced by the phase difference between modes $q_{2} = 3$ and $q_{1} = 1$ since the majority of energy is attributed to these modes^12^.

**Figure 1:**
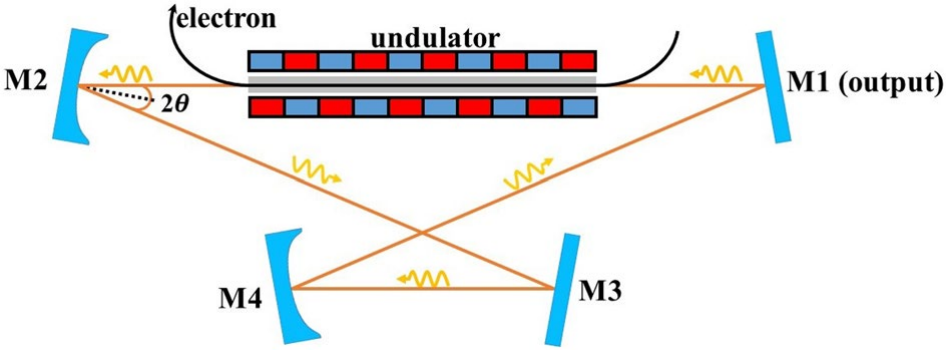
Schematic layout of the proposed scheme. The bow-tie cavity consists of two spherical mirrors denoted by M2 and M4, and two plane mirrors denoted by M3 and M1. In each roundtrip, after leaving the waveguide, the radiation field is initially focused by the spherical mirror M2, and passes through the flat mirror M3, then is focused by M4 and makes its way to the flat mirror M1 for the output coupling. Finally, the remaining radiation field returns to the waveguide, participating in subsequent interactions with the electron beam.

Conventionally, the resonant cavity of FELOs consists of two spherical mirrors and a rectangular waveguide in between. In this paper, we consider an improved operation mode to generate infrared radiation with high spatial and temporal quality, by using a four-mirror bow-tie resonator instead of a conventional two-mirror resonator. As a kind of ring cavity, the bow-tie cavity is constructed where light follows a closed path as illustrated in Fig. 1. There are two spherical mirrors and two plane mirrors in this device. After leaving the waveguide, the radiation field will be initially focused by a spherical mirror. When the radiation propagates within the bow-tie cavity, the transverse structure of the optical field undergoes changes, resulting in a Gaussian or approximate Gaussian distribution on the output coupling mirror. Nevertheless, the focusing effect of a spherical mirror is related to the off-axis angle, resulting in different focal strengths in the horizontal plane $\left( f_{H} \right)$ and vertical plane$\left( f_{V} \right)$^16,17^:

$$\begin{array}{l}f_{H}=\rho / 2 \cos \theta \\ f_{V}=\rho \cos \theta / 2\end{array}$$

where $\rho$ is the radius of curvature of the mirror and $\theta$ is the incident angle. It is inevitable that the system will introduce image astigmatism^18–20^ due to the the difference in focal lengths between the two planes. By implementing a compact structure, the incident angle can be reduced, which is an effective method for reducing astigmatism. For example, with an incident angle of about 5$^\circ$, the difference between the horizontal and vertical focal lengths is less than 0.8%, which can be considered negligible. Therefore, the four-mirror bow-tie cavity presented in this paper is often preferred in practical applications of ring laser cavity due to its low astigmatism^21^. Using the far-infrared oscillator of FELiChEM as an example, we demonstrate the performance of the proposed method. Through meticulous optimization, numerical simulation results reveal that the bow-tie cavity can reshape the transverse distribution of the radiation field at the output coupling mirror. The radiation field emerges an approximate Gaussian distribution in the transverse direction, which is beneficial to the output from the central coupling hole. This significantly enhances the output power, especially within the spectral gaps commonly encountered in conventional cavities, greatly facilitating users in conducting spectral scanning experiments.

## Simulation model

### FEL and optics code

A number of simulation codes of FEL oscillators have been developed in the literature, including codes that incorporate optical propagation algorithms into existing FEL simulation codes, as well as self-contained simulation codes for FEL oscillators^22–28^. In this paper, we propose utilizing an existing FEL simulation code to address the interaction within the undulator and establish a linkage with a dedicated code specifically designed for propagating the optical field across various resonator configurations. Within our simulation framework, the FEL code seamlessly transfers the optical field at the undulator exit to the optics code, which subsequently propagates the field throughout the resonator and returns it to the undulator entrance. Following this, the field is handed back to the FEL code for another pass through the undulator.

GENESIS^29^ is a three-dimensional simulation code that models the interaction between electrons and a co-propagating optical field through an undulator line. In an oscillator FEL with a waveguide, the electromagnetic wave is restricted by the metal pipe wall as the boundary. The radiation field that propagates inside the waveguide can be decomposed into different waveguide modes, not like a light flux in a free space FEL. Comparing the situation in the waveguide and free space, the longitudinal dependence of the electron beam and radiation field, including the resonant frequency and group velocity, is approximately the same^14^. Despite this, the waveguide FEL is distinguishable from its free-space counterpart in that its transverse field evolution is influenced by the waveguide itself^30^. Considering the conductive boundary conditions, a modified GENESIS code has been developed for the FEL using a rectangular waveguide^31^.

The optics propagation code (OPC)^32^ can be employed to simulate oscillators or propagate an optical field from the end of the undulator line to a specific point of interest. OPC propagates the optical field either using the Fresnel diffraction integral or the spectral method in paraxial approximation using fast discrete Fourier transforms (FFT). Additionally, a modified Fresnel diffraction integral^17,33^ is provided, which enables the utilization of Fast Fourier Transforms (FFTs) along with an expanding grid to define the optical field. This method is commonly employed when significant optical field diffraction occurs. The current version of OPC incorporates a range of optical elements, including mirrors, lenses, and round or rectangular diaphragms. Lenses or mirrors will generates a phase shift to the optical field. This is done by multiplying the optical field by $e^{-i u(x, y)}$, where *u*(*x*, *y*) is the local phase shift in the transverse plane^34^. The spherical mirror can be modeled as a thin lens with focal strength $f = \rho /2$^17^, where $\rho$ is the radius of curvature of the mirror. A thin lens is modeled as^34^:

$$u(x, y) = \frac{k_{0}(x^{2}+y^{2})}{2f}$$

where $k_{0} = \frac{2\pi }{\lambda _{0}}$, $\lambda _{0}$ is the free-space wavelength. These elements can be combined to create complex optical components. For instance, by combining a mirror with a hole element, it becomes possible to model the extraction of radiation from a resonator through a hole in one of the mirrors^35^. It should be noted that the default optical elements employed in OPC are regarded as ideal thin lenses or spherical mirrors. Nevertheless, OPC also offers support for more intricate phase masks created using Zernike polynomials^33^. These polynomials are utilized to generate a phase difference $d\theta$ defined on a transverse plane, which is then applied to the optical field. Within OPC this application is expressed in the following equations:

$$d \theta =A_{n m} R_{n}^{|m|}(r) \times \left\{ \begin{array}{ll}\cos (m \phi ) &{} m \ge 0 \\ \sin (m \phi ) &{} m<0\end{array}\right.$$

where $R_{n}^{|m|}$ is the circle polynomial of order (*n*, *m*)^33^, *r* is the scaled radial distance, $r=\sqrt{x^{2}+y^{2} } /r _{c}$ with $r _{c}$ being the characteristic length, $\phi$ is the angle $\tan ^{-1} (y/x)$ and $A_{nm}$ is the amplitude of the polynomial. These polynomials define a phase mask applied to the optical field at the position of the corresponding optical component. An application of these Zernike polynomials is to synthesize novel optical components. For instance, by superimposing Zernike polynomials such as *m*, *n* = 2, 2 and *m*, *n* = 0, 2, with the appropriate amplitudes, one can effectively generate a cylindrical lens with a certain focal strength^36^. Through the combination of two cylindrical mirrors, focusing in the horizontal direction and vertical direction respectively, and applying appropriate focal strengths to these mirrors, one can accurately model the performance of a spherical mirror with a non-zero incident angle^36^. Here, we employ Zernike polynomials to model the intricate behavior of spherical mirrors in the bow-tie cavity. By combining the modified GENESIS code with the OPC code, the waveguide FEL with a bow-tie structure can be effectively modelled.

### Simulation parameters

**Figure 2:**
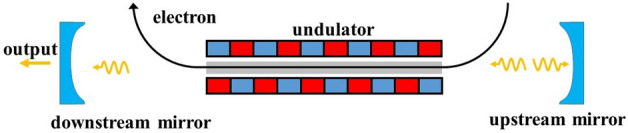
FELiChEM resonant cavity diagram. The curvature radius of each mirror is 3.018 m and the distance between cavity mirrors is 5.04 m. The laser output coupling is achieved by a hole of 2.0 mm radius in the center of the downstream mirror. The rectangular waveguide length of 2.24 m only fits the undulator section, and the rest of resonant cavity is in free space.

The simulations were carried out using the far-infrared FEL oscillator at the FELiChEM facility^11^. The electron beam is generated by a pulser-gated thermionic gun and then is accelerated to the energy range of 12 MeV to 60 MeV with a micropulse length of about 4.5 ps. The repetition frequency of the electron microbunch can be set at 119 MHz or 59.5 MHz, while the cavity length of the far-infrared oscillator is 5.04 m. As shown in Fig. 2, the two-mirror optical cavity is currently designed as a near-concentric resonator and a planar undulator is located in the center of the cavity to wiggle the electron beam and adjust the wavelength of the FEL. At the present stage, the radiation field is confined within a partially rectangular waveguide in the optical cavity, with the size of 30 mm × 16 mm. Nevertheless, we have observed that under this waveguide size, boundary losses lead to a significant reduction in output power at long wavelengths. In the example presented here, a redesigned waveguide size of 30 mm × 20 mm has been selected. Apart from the rectangular waveguide dimensions, the simulation parameters are the same as the actual ones of the FELiChEM far-infrared FEL^37^. The parameters of the conventional cavity are summarized in Table 1. The undulator module of length 2.24 m has 40 periods of wavelength $\lambda _{u}$ = 5.6 cm. The average electron beam energy is 15 MeV, resulting in a resonant radiation wavelength ranging from 50 to 200 μm. An coupling hole with the radius of 2 mm is used in the oscillator (in the downstream mirror). The total roundtrip loss is therefore 1.99% since both mirrors have a loss of 1.0%.

The bow-tie cavity (as shown in Fig. 1) consists of two plane mirrors and two spherical mirrors. For an effective collision between electron and photon, it is imperative to select the dimensions of the four-mirror optical cavity based on the electron bunch repetition rate, ensuring that the total optical path length of the cavity is precisely 10.08 m. In the bow-tie cavity, distribution of the radiation field is determined by a number of factors. The choice of the radius of curvature of the two spherical mirrors and the distance between each optical element is of critical importance.

**Figure 3:**
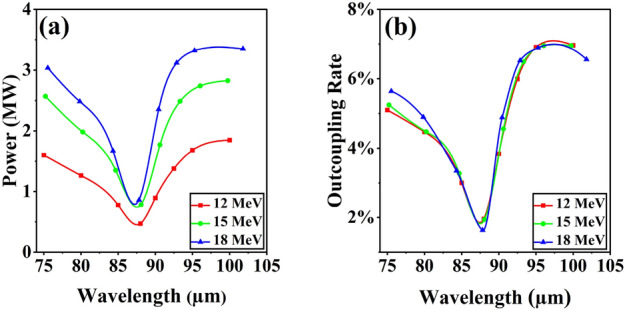
(**a**) The radiation output power and (**b**) outcoupling rate in the conventional cavity with different electron beam energies.

**Figure 4:**
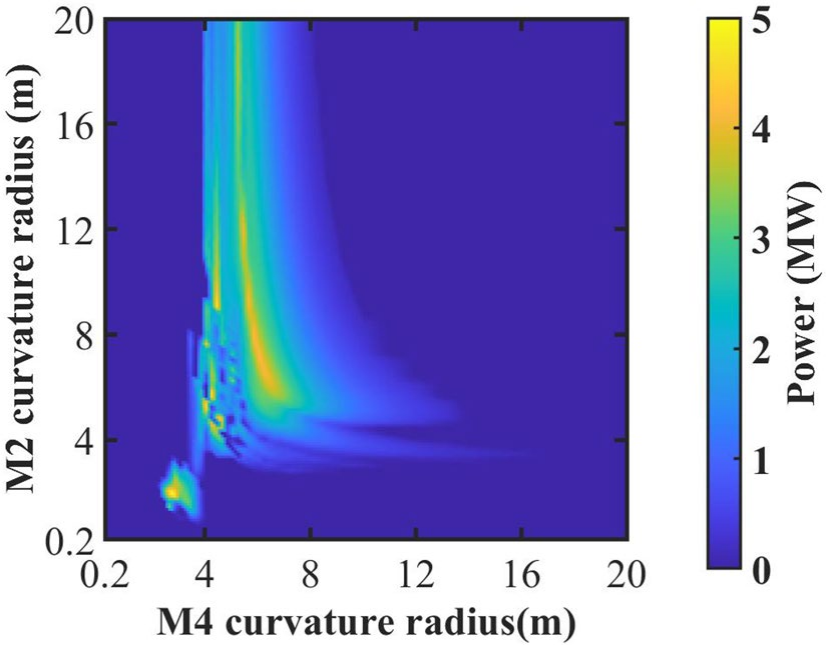
The output power as a function of the curvature radius of spherical mirrors M2 and M4, for the wavelength of 88 μm.

As shown in Fig. 3, a distinct spectral gap is consistently observed at about 88 μm, which is close to the theoretical value of 89.2 μm. Regardless of the adjustments made to the beam parameters, this phenomenon persists in the conventional cavity. Therefore, it is essential and reasonable for us to optimize at the radiation wavelength of 88 μm, with the expectation of significantly increasing the outcoupling power. We keep the average electron beam energy at 15 MeV and the total optical path of each pass in the bow-tie cavity is fixed at 10.08 m. Moreover, the incident angle should not be too large in order to reduce image astigmatism. As shown in Fig. 1, the bow-tie cavity has an intricate structure composed of two similar triangular shapes. With a fixed total cavity length and a small incident angle, the adjustment range for the distance between M2 and M3 is limited. For this reason, we keep the length between M2 and M3 at 2.54 m, with each mirror having an incident angle of $5.09^\circ$. It should be noted that the distance between M2 and M3 is the same as that between M4 and M1. Therefore, excluding the aforementioned lengths and the undulator length (2.24 m), the remaining optical path length of the bow-tie cavity is 2.76 m. At the beginning, we set the distance between the undulator and M2 as well as the distance between the undulator and M1 to 1.0 m. The roundtrip number is fixed at 300 to make sure the intracavity power can reach saturation. As shown in Fig. 4, the saturated output power is plotted as a color contour plot when the curvature radius of spherical mirrors M2 and M4 is changed. Simulation results show that the output power increases significantly when the curvature radius of M2 is around 2.0 m and M4 is around 2.6 m. Furthermore, one can find that the output power remains a relatively high level when the curvature radius of M2 is about 7.2 m, and for M4, it is around 6.0 m. It should be pointed out that the focusing effect in the bow-tie cavity is determined by the combination of the curvature radii of spherical mirrors M2 and M4. By employing different combinations of M2 and M4, as the radiation field passes through different positions within the bow-tie cavity, it will exhibit distinctly different beam sizes. At the entrance of waveguide, the radiation field with a relatively small beam size will experience less boundary loss from the waveguide, facilitating the achievement of higher output power.

Another crucial aspect is to adjust the distances between various components to achieve a smaller beam size at the entrance of waveguide. The distances between mirror M1 and the undulator, as well as between mirror M2 and the undulator, should both be at least around 0.6 m owing to magnetic dipoles are required to bend the electron bunch during its entry and exit from the undulator. With careful optimization, we can achieve a notable increase in output power from about 4.6 MW to 6.0 MW. The simulation results are obtained under the specific configuration with the curvature radius of 2.0 m for M2 and 2.6 m for M4. After optimization, the essential parameters of the bow-tie cavity used in the simulations are listed in Table 2. For the purpose of comparison, the parameters of the electron beam and the undulator in the bow-tie cavity are identical to those in the conventional cavity. We only change the curvature radius of reflective mirrors, while maintaining all other parameters in line with conventional cavity mirrors. However, it is important to note that there are four mirrors in a bow-tie cavity, which results in a round trip loss from the mirror absorption of 3.94%, approximately twice as much as that of a conventional cavity.

**Table 1:** FEL system parameters of the conventional cavity.

Parameter	Value	Unit
Beam energy	15	MeV
Energy spread (rms)	1.00	%
Peak current	94	A
Normalized emittance	30	$\mathrm {mm \cdot mrad}$
Undulator parameter (rms)	0.73–2.26	–
Undulator period	5.6	cm
Number of periods	40	–
Resonator length	5.04	m
Waveguide size $\left( a\times b \right)$	30 × 20	$\mathrm {mm \times mm}$
Curvature radius of mirror	3.018	m
Radius of coupling hole	2.00	mm
Reflectivity of mirror	99.00	%
Diameter of mirror	8.00	cm

**Table 2:** Summary of the bow-tie cavity parameters used in the simulations.

Parameter	Value	Unit
The total path length of cavity	10.08	m
The length between undulator and M2	1.12	m
The length between M2 and M3	2.54	m
The length between M3 and M4	0.88	m
The length between M4 and M1	2.54	m
The length between M1 and undulator	0.76	m
The incident angle ($\theta$)	5.09	°
The curvature radius of M2	2.00	m
The curvature radius of M4	2.60	m
Radius of coupling hole	2.00	mm
Reflectivity of mirror	99.00	%
Diameter of mirror	8.00	cm

## Results and discussions

**Figure 5:**
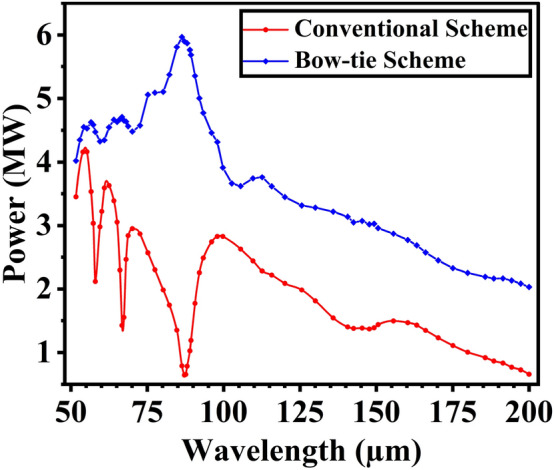
Comparison of the output power between the conventional cavity (red) and the proposed bow-tie scheme (blue).

**Figure 6:**
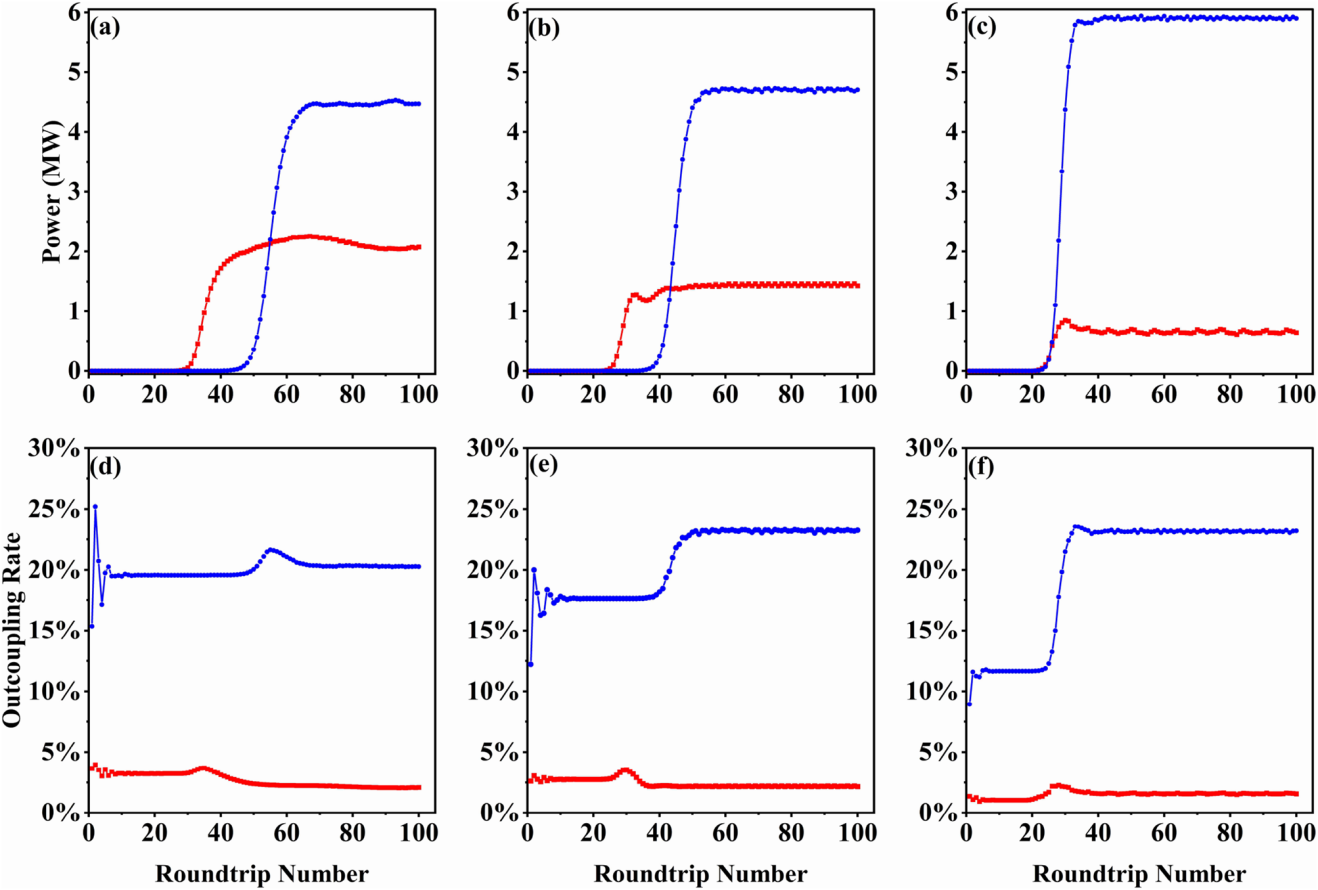
Comparison of the output power and the outcoupling rate between the conventional cavity (red) and the proposed bow-tie scheme (blue). (**a**) and (**d**) are corresponding to 58 μm, (**b**) and (**e**) are corresponding to 67 μm and (**c**) and (**f**) are corresponding to 88 μm.

For the purpose of comparison, we carried out numerical simulations for both conventional cavity and the proposed scheme. The relationship between output power and radiation wavelength is shown in Fig. 5. The red curve corresponds to the simulation results of the conventional scheme, and the blue curve corresponds to the bow-tie cavity. In the conventional cavity, apart from a significant spectral gap at 88 μm, there are also spectral gaps at 58 and 67 μm, although they are not as severe as the one at 88 μm. In the bow-tie cavity, the optical field leaves the waveguide, is initially focused by spherical mirrors, and then reaches M1 for central hole coupling, eliminating the spectral gap phenomenon and enhancing power output. When the spectral gap is most noticeable at wavelength 88 μm, the radiation power increases from about 0.6 MW to about 6 MW. Additionally, the output power also increases significantly at wavelengths of 58 μm and 67 μm. It is worth noting that at longer wavelengths, the radiation field passes through the waveguide only once per roundtrip, thereby reducing boundary losses. Evidently, at the radiation wavelength of 200 μm, the output power increases from 0.6 MW to 2.0 MW. Figure 6 shows the growth of output power and outcoupling rate as a function of the cavity roundtrip number. After reaching saturation, the output power of the conventional cavity remains below 2.5 MW, with an outcoupling rate below 2.5%. In contrast, the bow-tie cavity not only experiences a substantial increase in output power but also attains an outcoupling rate exceeding 20%.

**Figure 7:**
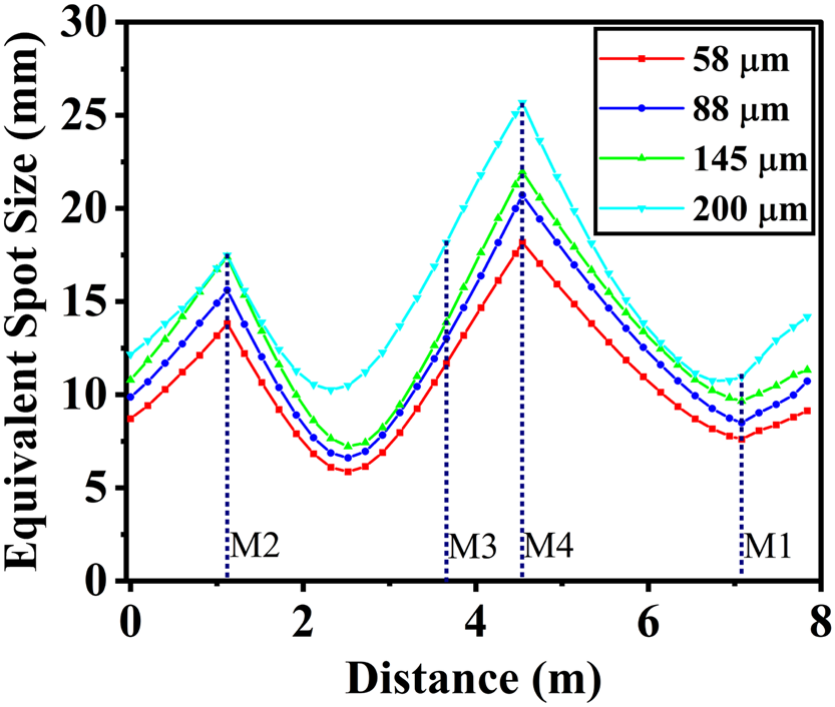
After saturation, the corresponding spot radius of the radiation field varies within the bow-tie cavity during one roundtrip..

After saturation, with the wavefront propagation code OPC, the corresponding spot radius of the radiation field (equivalent spot radius defined in OPC code) as it propagates through different positions in the bow-tie cavity are shown in Fig. 7 to clarify the gradient variation. As the optical field propagates from the undulator to mirror M2, the spot radius gradually increases. When the radiation pulse reaches the spherical mirror M2, it undergoes focusing, resulting in a reduction in the spot radius. It should be noted that the plane mirror M3 cannot modify the wavefront of radiation fields, which means that the radiation spot size will continue to diverge. After passing through the spherical mirror M4, the transverse distribution of radiation fields is focused and reaches the flat mirror M1 for central hole coupling output. As the radiation wavelength increases, the spot radius in the resonant cavity experience an expansion due to heightened diffraction effects. In comparison to shorter wavelengths, the more pronounced diffraction at longer wavelengths results in a broader transverse distribution of the optical field. Furthermore, it should be noted that the peak power density of intracavity radiation sharply decreases since a portion of the radiation pulse is coupled out through the central hole at mirror M1. As a result, numerical statistics about the spot radius after mirror M1 will be overestimated.

**Figure 8:**
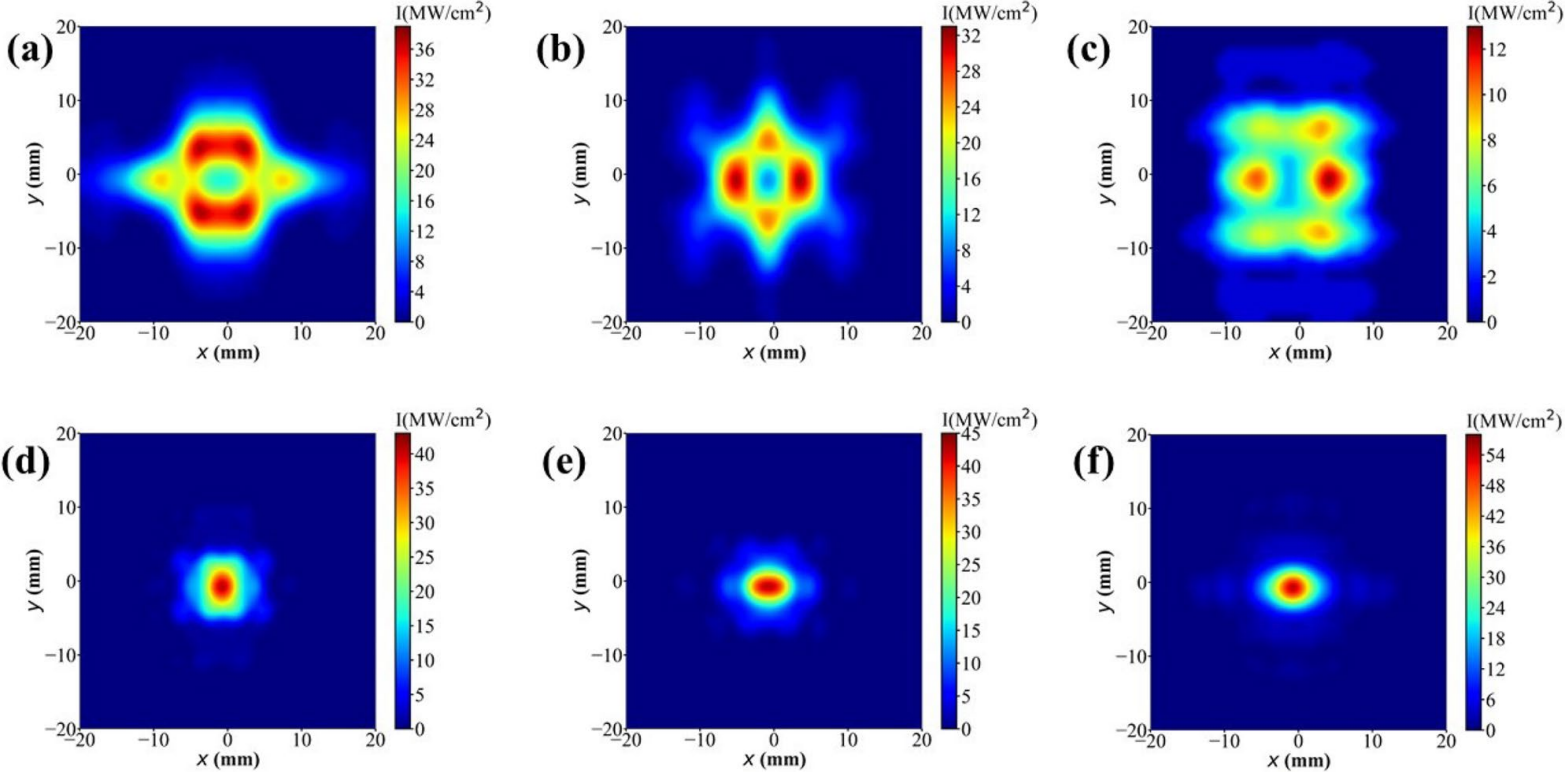
After saturation, transverse profiles of the radiation field on the coupling output mirror. The conventional cavity is depicted in the upper image, while a bow-tie cavity is depicted in the lower image. In addition, (**a**) and (**d**), (**b**) and (**e**), (**c**) and (**f**) represent 58 μm, 67 μm and 88 μm, respectively.

**Figure 9:**
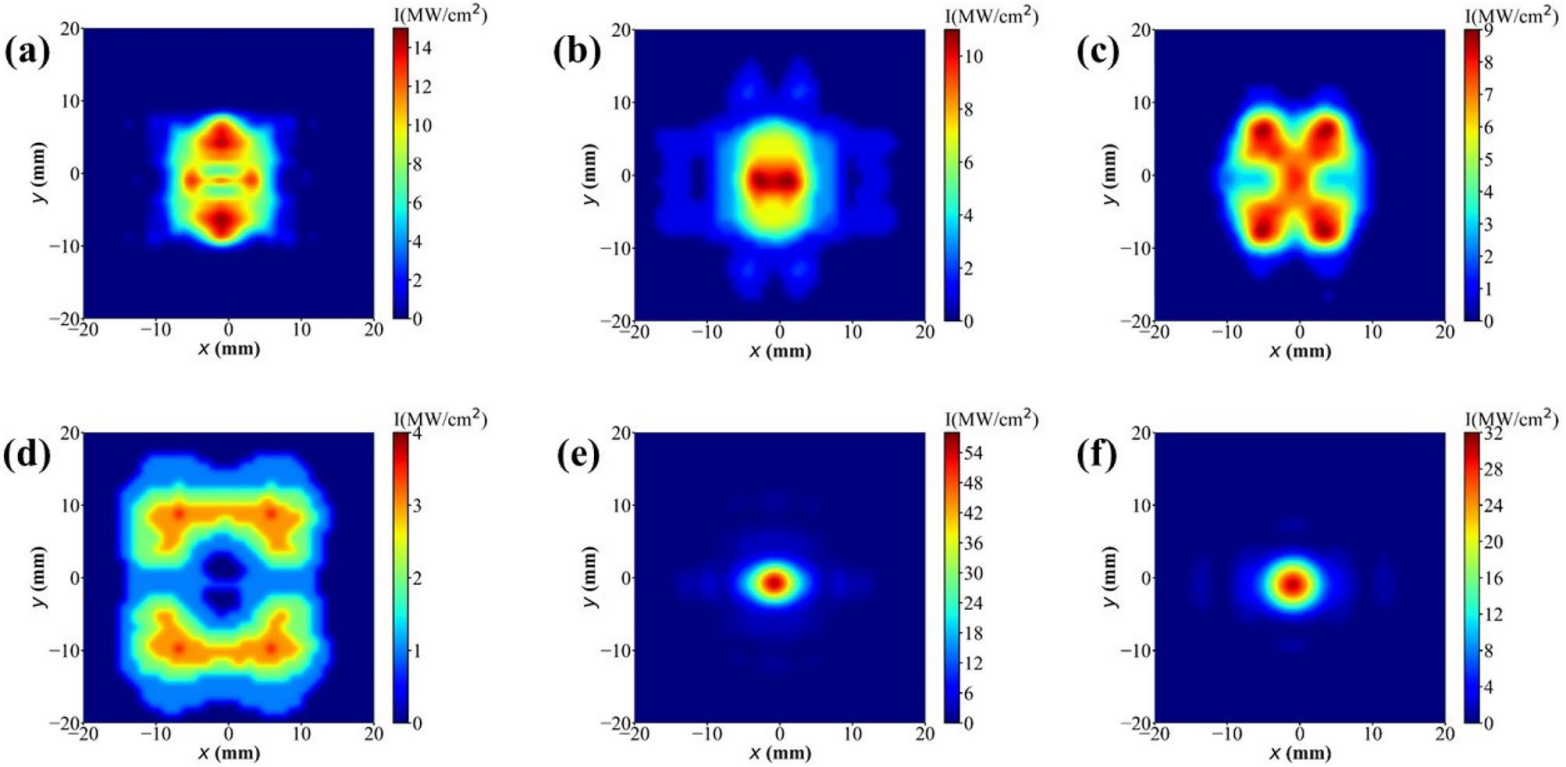
After saturation, transverse profiles of the radiation field within the bow-tie cavity for the radiation wavelength of 88 μm, including (**a**) undulator exit, (**b**) mirror M2, (**c**) mirror M3, (**d**) mirror M4, (**e**) outcoupling mirror M1 and (**f**) undulator entrance.

The transverse profiles of the radiation field on the coupling output mirror are illustrated in Fig. 8. In a conventional cavity, the presence of a waveguide results in a transverse field distribution deviating significantly from the Gaussian shape at specific wavelengths. The optical waveguide modifies the internal optical mode of the resonant cavity, leading to a substantial reduction in optical field intensity at the center of the output mirror. Consequently, only a limited amount of laser power can be coupled out through the central hole. In Fig. 9 the transverse power profiles, with their peak power densities, are plotted after saturation at different points within the bow-tie cavity. The simulation is conducted at the radiation wavelength of 88 μm, where the spectral gap phenomenon is most pronounced. From Fig. 9, one can observe the transverse distribution of the radiation pulse varies at different positions within the bow-tie cavity. Since the radiation pulse is not coupled out immediately after leaving the waveguide, the transverse distribution of the radiation field will change as it passes through spherical mirrors. As a result, the transverse profiles of radiation field exhibits an approximate Gaussian distribution at the coupling output mirror M1. In this way, the bow-tie cavity offers an advantage over conventional resonant cavities in that it can eliminate the spectral gap phenomenon introduced by waveguide FELs.

## Conclusions

In conclusion, a novel technique is proposed for generating intense infrared radiation pulses by fully exploiting the advantages of waveguide FELs. The cavity consists of four separate high-reflectivity mirrors arranged in a bow-tie configuration. In waveguide FELs with conventional designs, the FEL power is relatively low at certain wavelengths due to the spectral gap phenomenon. Spectral gaps will impose substantial adverse effects on some users, particularly those engaged in spectral scanning experiments, which is a crucial issue that needs to be urgently solved. Compared with a conventional cavity, the proposed scheme offers the unique advantage that radiation fields are not coupled to output immediately after leaving the waveguide. The bow-tie resonant cavity offers enhanced flexibility in designing the focusing system, and the radiation fields pass through the waveguide only once per roundtrip, thereby avoiding its potential adverse effects. In a bow-tie resonant cavity, the radiation beam is first focused by spherical mirrors, resulting in a Gaussian or close to Gaussian distribution of radiation fields on the coupling mirror after propagating a fixed distance. As a result, the proposed method can effectively eliminate the spectral gap phenomenon. A simulation based on the far-infrared parameters of FELiChEM was performed as an example. The electron beam and undulator parameters used in the bow-tie cavity are identical to those used in the traditional cavity. According to numerical simulations, the bow-tie cavity can mitigate the detrimental effects of spectral power gaps on waveguide FELs, enhance coupling output power, and effectively improve infrared FEL performance as compared to the traditional resonant cavity.

## Data Availability

The data supporting the conclusions of this study are available from the corresponding author upon reasonable request.
